# Improved Dehydrogenation Properties of LiAlH_4_ by Addition of Nanosized CoTiO_3_

**DOI:** 10.3390/nano12213921

**Published:** 2022-11-07

**Authors:** Nurul Amirah Ali, Muhammad Amirul Nawi Ahmad, Muhammad Syarifuddin Yahya, Noratiqah Sazelee, Mohammad Ismail

**Affiliations:** Energy Storage Research Group, Faculty of Ocean Engineering Technology and Informatics, Universiti Malaysia Terengganu, Kuala Nerus 21030, Malaysia

**Keywords:** lithium aluminium hydride, hydrogen storage, additives, cobalt titanate

## Abstract

Despite the application of lithium aluminium hydride (LiAlH_4_) being hindered by its sluggish desorption kinetics and unfavourable reversibility, LiAlH_4_ has received special attention as a promising solid-state hydrogen storage material due to its hydrogen storage capacity (10.5 wt.%). In this work, investigated for the first time was the effect of the nanosized cobalt titanate (CoTiO_3_) which was synthesised via a solid-state method on the desorption behaviour of LiAlH_4_. Superior desorption behaviour of LiAlH_4_ was attained with the presence of a CoTiO_3_ additive. By means of the addition of 5, 10, 15 and 20 wt.% of CoTiO_3_, the initial desorption temperature of LiAlH_4_ for the first stage was reduced to around 115–120 °C and the second desorption stage was reduced to around 144–150 °C, much lower than for undoped LiAlH_4_. The LiAlH_4_-CoTiO_3_ sample also presents outstanding desorption kinetics behaviour, desorbing hydrogen 30–35 times faster than undoped LiAlH_4_. The LiAlH_4_-CoTiO_3_ sample could desorb 3.0–3.5 wt.% H_2_ in 30 min, while the commercial and milled LiAlH_4_ desorbs <0.1 wt.% H_2_. The apparent activation energy of the LiAlH_4_-CoTiO_3_ sample based on the Kissinger analysis was decreased to 75.2 and 91.8 kJ/mol for the first and second desorption stage, respectively, lower by 28.0 and 24.9 kJ/mol than undoped LiAlH_4_. The LiAlH_4_-CoTiO_3_ sample presents uniform and smaller particle size distribution compared to undoped LiAlH_4_, which is irregular in shape with some agglomerations. The experimental results suggest that the CoTiO_3_ additive promoted notable advancements in the desorption performance of LiAlH_4_ through the in situ-formed AlTi and amorphous Co or Co-containing active species that were generated during the desorption process.

## 1. Introduction

Hydrogen is a clean and renewable energy carrier and has become the most preferred alternative to fossil fuels for the future energy system. The advancement of secure and effective solid-state hydrogen storage that operates at low temperature was the focus in developing practical hydrogen as a potential energy carrier, offering a high capacity of hydrogen and excellent reversibility. Recently, alanates have been considered favourable materials for solid-state hydrogen storage with high hydrogen capacity and operating at low temperatures compared to other hydrides [1,2]. Among the alanates, lithium aluminium hydride (LiAlH_4_) has attracted considerable research interest. LiAlH_4_ offers 10.5 wt.% hydrogen content [3]. Below 250 °C, LiAlH_4_ is able to liberate a theoretical capacity of 7.9 wt.% H_2_ [4,5]. Basically, LiAlH_4_ decomposes to LiH and Al in a three-step reaction as follows [6,7]:

3LiAlH_4_ → Li_3_AlH_6_ + 2Al + 3H_2_ (1)

(occurs at 150–175 °C, releasing 5.3 wt.% H_2_)

Li_3_AlH_6_ → 3LiH + Al + 3/2H_2_ (2)

(occurs at 180–220 °C, releasing 2.6 wt.% H_2_)

3LiH + 3Al → 3LiAl + 3/2H_2_ (3)

(occurs at >400 °C, releasing 2.6 wt.% H_2_)

However, inadequate reversibility and unfavourable desorption kinetics of LiAlH_4_ obstruct its progression to practical utilisation as a solid-state hydrogen storage medium [8]. Furthermore, the 2.6 wt.% H_2_ in LiH as in Equation (3) is impracticable for applications owing to its high decomposition temperature (higher than 400 °C) [9]. To overcome these problems, several techniques have been introduced to accelerate the desorption behaviour of LiAlH_4_—for example, the use of the nanoconfinement method and high energy ball milling [10,11,12,13], the usage of additives [14,15,16,17] and destabilisation with other hydrides [18,19,20].

The technique of high energy ball milling may exert a colossal effect on the behaviour of solid-state hydrogen storage materials by reducing the particle size that results in a larger surface area, hence shortening the distance of hydrogen diffusion [21,22]. The presence of additives, especially metal oxides, leads to a significant improvement, with a lower decomposition temperature and faster kinetic performance of LiAlH_4_ due to enhanced catalytic support [23,24,25,26]. For instance, Li et al. [27] demonstrated satisfactory desorption behaviour of LiAlH_4_ with the addition of NiCo_2_O_4_. The LiAlH_4_-NiCo_2_O_4_ sample could release 4.95 wt.% H_2_ at 130 °C in 150 min, while undoped LiAlH_4_ only releases <1.0 wt.% H_2_ within the same period. Then, Sulaiman et al. [28] showed that LiAlH_4_ added with SrFe_12_O_19_ began to decompose at temperatures of 80 °C (first stage) and 130 °C (second stage), which are lower than undoped LiAlH_4_. They also observed the formation of Fe, LiFeO_2_, Sr and Sr-containing species during the heating process, which are advantageous in boosting the desorption properties of LiAlH_4_. Furthermore, Li et al. [29] presented a study on the influence of CoFe_2_O_4_ on the desorption of LiAlH_4_. Surprisingly, the performance of LiAlH_4_ was significantly enhanced with rapid hydrogen desorption and low dehydrogenation activation energy. The LiAlH_4_-CoFe_2_O_4_ sample could desorb 6.8 wt.% H_2_ in 160 min, 6.1 wt.% higher than undoped LiAlH_4_. Moreover, the activation energy of the LiAlH_4_-CoFe_2_O_4_ composites decreased by 42.4 kJ/mol (first stage) and 86.1 kJ/mol (second stage) compared to pure LiAlH_4_. The enhanced performance of the LiAlH_4_-CoFe_2_O_4_ composite was attributed to the formation of Fe and Co species due to the reaction between LiAlH_4_ and CoFe_2_O_4_. Then, another study revealed that the addition of TiO_2_ could notably downshift the decomposition temperature of LiAlH_4_ to 60 °C [30]. Desorption kinetics performance at 100 °C demonstrated that a TiO_2_-doped LiAlH_4_ composite could desorb 5.2 wt.% H_2_ in 30 min, whereas the commercial LiAlH_4_ desorbed only <0.2 wt.% H_2_ in the same time but at a higher temperature (120 °C). The addition of TiO_2_ offers significant enhancement of the performance of LiAlH_4_ that may be ascribed to the introduction of a high density of defects on the surfaces of the TiO_2_ particles throughout the ball milling process.

Inspired by the superior performance of the metal oxide additives, it is motivating to discover the consequence of another metal oxide on the desorption behaviour of LiAlH_4_. The metal oxide additives may present outstanding performance compared to metal because of the bulk of oxides and the transition metal ions on the surface that may encounter different crystal fields due to the lack of ions at the surface of these oxides. This allows the electronic 3d state of the ions to be separated and is beneficial for the catalytic activity of transition metal oxides in the adsorption of gas molecules [31]. In this study, nanosized cobalt titanate (CoTiO_3_) as an additive was added to LiAlH_4_ by means of a ball milling process to ameliorate the desorption behaviour of LiAlH_4_. Nanosized approaches are favourable in decreasing the materials’ particle size and reducing the hydrogen diffusion distance, accelerating the rate of hydrogen diffusion and nucleation sites for the desorption process [32]. Moreover, it is believed that nanosized CoTiO_3_ could provide a superior performance of LiAlH_4_ with low hydrogen release temperature and faster kinetics. Herein, different amounts of CoTiO_3_ were milled together with LiAlH_4_ to examine the desorption behaviour of LiAlH_4_ as well as its kinetics behaviour. Moreover, the possible catalytic mechanism of the desorption process for this system has been discussed in depth.

## 2. Materials and Methods

Without any prior processing, LiAlH_4_ (95% pure) from Sigma Aldrich was used. The nanosized CoTiO_3_ was synthesised via the solid-state method, as explained in our previous work [33]. Then, various amounts of CoTiO_3_ (5 wt.%, 10 wt.%, 15 wt.% and 20 wt.%) were doped with LiAlH_4_ to study its influence on the desorption behaviour of LiAlH_4_. The composite was milled together at a speed of 400 rpm for one hour using a planetary ball mill (NQM-0.4).

A Sievert-type pressure-composition-temperature apparatus from the Advanced Materials Corporation was operated to carry out the hydrogen desorption kinetics experiment and the temperature-programmed desorption (TPD). The TPD test was carried out in a vacuum chamber and heated to 250 °C for the purpose of finding the initial desorption temperature. Experiments for the isothermal desorption tests were carried out at 90 °C and 1.0 atm H_2_ pressure. Using a Mettler Toledo TGA/DSC 1, differential scanning calorimetry (DSC) measurements were carried out to analyse the thermal characteristics and determine the activation energy. For each characterisation, 6–8 mg of the sample was loaded in a crucible and heated to 300 °C with a flow of argon (50 mL/min) at several heating rates (15 °C/min, 20 °C/min, 25 °C/min and 30 °C/min).

The phase structure of the LiAlH_4_-CoTiO_3_ sample after milling and after the desorption sample was examined using a Rigaku MiniFlex X-ray diffraction (XRD) system with Cu Kα radiation. The sample was tested at a rate of 2.00°/min over diffraction angles of 20° to 80°. The sample’s morphology and microstructure were then examined with a scanning electron microscope ((SEM; JEOL, Akishima, Tokyo, Japan) (JSM-6350LA)). The Fourier transform infrared (FTIR) spectrometer was tested in the range of 700 and 2000 cm^−1^ using IR Shimadzu Tracer-100.

## 3. Results and Discussions

**Figure 1:**
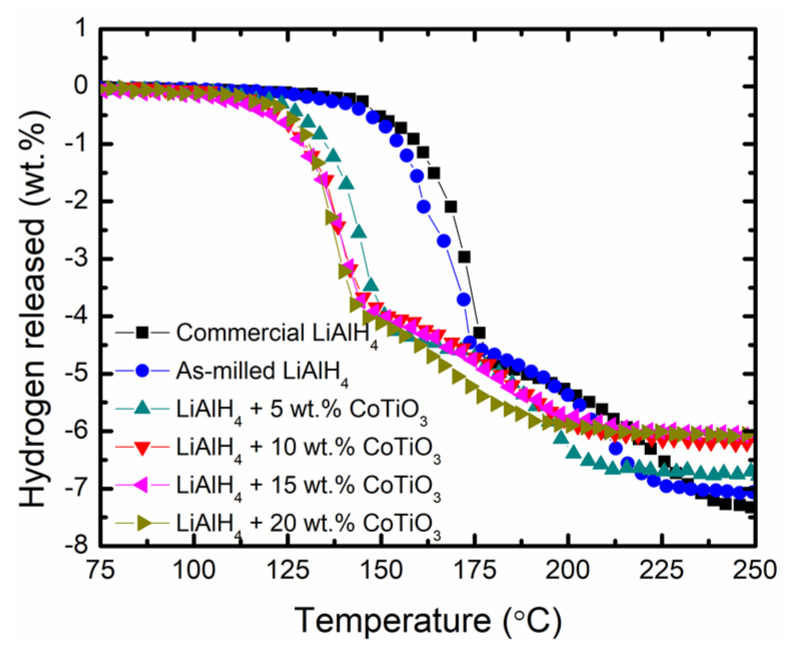
TPD profile of commercial LiAlH_4_, as-milled LiAlH_4_ and LiAlH_4_ + *x*CoTiO_3_ (*x* = 5, 10, 15 and 20 wt.%).

The influence of CoTiO_3_ on the initial desorption temperature of LiAlH_4_ was characterised by the TPD experiment, as presented in Figure 1. By referring to the figure, the addition of CoTiO_3_ results in superior desorption performance of LiAlH_4_. Commercial LiAlH_4_ begins to release hydrogen at 145 °C (first stage) and 175 °C (second stage) releasing around 7.4 wt.% H_2_. After 1 h of milling, the temperature to release hydrogen was similar to the commercial LiAlH_4_, demonstrating that the milling process had a negligible influence on the desorption temperature of LiAlH_4_. Contrarily, by adding different amounts of CoTiO_3_ (5, 10, 15 and 20 wt.%), the hydrogen release initiates at a much lower temperature than commercial and milled LiAlH_4_. The LiAlH_4_ + 5 wt.% CoTiO_3_ sample began to release hydrogen at 120 °C (first stage) and 150 °C (second stage), releasing 6.9 wt.% H_2_. Furthermore, increasing the amount of CoTiO_3_ to 10 wt.% resulted in a decrease in hydrogen release temperature to 115 °C (first stage) and 145 °C (second stage), releasing 6.2 wt.% H_2_. Adding 15 wt.% of CoTiO_3_ resulted in a similar temperature for hydrogen release as the LiAlH_4_ + 10 wt.% CoTiO_3_ sample. For the LiAlH_4_ + 20 wt.% CoTiO_3_ sample, hydrogen release occurs at 118 °C (first stage) and 144 °C (second stage), releasing 6.0 wt.% H_2_. The hydrogen release temperature for all of the CoTiO_3_-doped LiAlH_4_ samples was much lower for than the undoped LiAlH_4_. Notably, with the amount of hydrogen released being slightly reducing compared to the undoped LiAlH_4,_ the presence of CoTiO_3_ provides positive contributions in lowering the temperature for the LiAlH_4_ to release hydrogen. The amount of hydrogen released for all of the CoTiO_3_-doped LiAlH_4_ samples was slightly reduced compared to undoped LiAlH_4_ due to the deadweight of CoTiO_3_ that does not hold any hydrogen [10,34].

**Figure 2:**
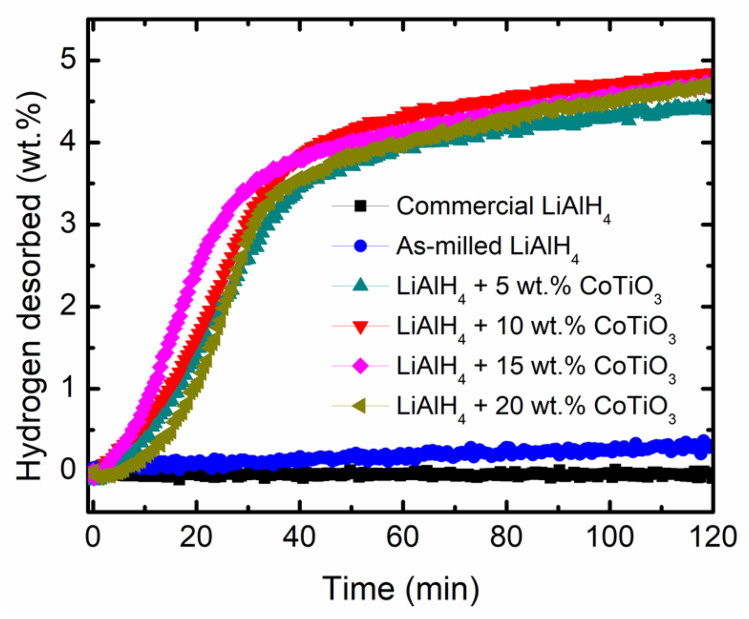
The desorption kinetic curve of commercial LiAlH_4_, as-milled LiAlH_4_ and LiAlH_4_ + *x*CoTiO_3_ (*x* = 5, 10, 15 and 20 wt.%).

Additionally, Figure 2 demonstrates the desorption kinetic performance of LiAlH_4_ with and without CoTiO_3_ at 90 °C. As shown in Figure 2, the LiAlH_4_ + *x*CoTiO_3_ (*x* = 5, 10, 15 and 20 wt.%) releases a large amount of hydrogen in a short period of time. Within 30 min, the LiAlH_4_-CoTiO_3_ sample releases 3.0–3.5 wt.% H_2_. However, the undoped LiAlH_4_ shows sluggish kinetics, with the ability to release only <0.1 wt.% H_2_ within the same period. The addition of CoTiO_3_ results in accelerated desorption kinetics which are 30–35 times faster than undoped LiAlH_4_. The rapid hydrogen release from the LiAlH_4_-CoTiO_3_ sample could be related to the creation of surface defects and active materials as a result of a reaction between LiAlH_4_ and CoTiO_3_. Therefore, it can be concluded that the kinetic behaviour of LiAlH_4_ can be enhanced by introducing CoTiO_3_. Considering the initial desorption temperature and the desorption kinetics performance, the LiAlH_4_ + 10 wt.% CoTiO_3_ sample is selected to further examine the effect of CoTiO_3_ on the desorption behaviour of LiAlH_4_.

**Figure 3:**
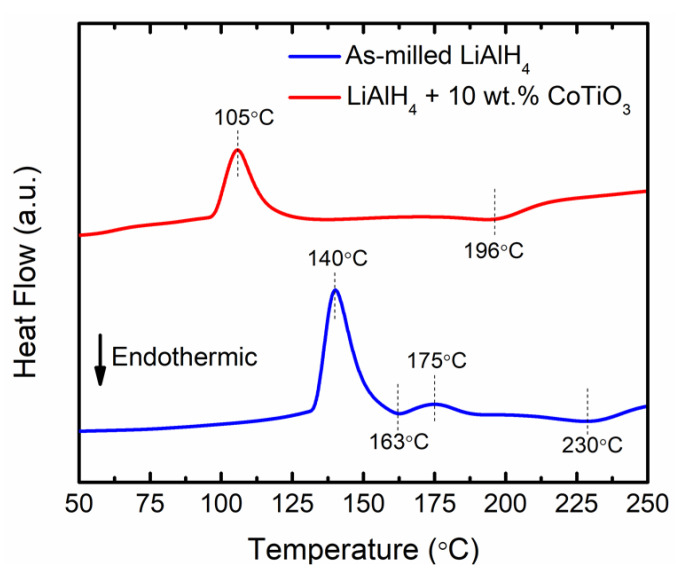
DSC profile of as-milled LiAlH_4_ and LiAlH_4_ + 10 wt.% CoTiO_3_ (heating rate: 15 °C/min).

The effect of CoTiO_3_ on the thermal behaviour of LiAlH_4_ was characterised by DSC, as indicated in Figure 3. By referring to Figure 3, the thermal properties of as-milled LiAlH_4_ consist of four peaks attributed to the two endothermic and two exothermic peaks. The first exothermic peak (140 °C) was due to the interaction of hydroxyl impurities at the surface of LiAlH_4_, while the first endothermic peak (163 °C) is attributed to the melting of LiAlH_4_. The second exothermic peak (175 °C) is ascribed to the process of LiAlH_4_ decomposing to Li_3_AlH_6_ and Al, while the second endothermic peak (230 °C) relates to the process of Li_3_AlH_6_ decomposing to LiH and Al. Upon the addition of CoTiO_3_, the number of peaks was decreased to two, namely one endothermic peak and one exothermic peak, respectively, that occur at a lower temperature than for milled LiAlH_4_. The exothermic peak (LiAlH_4_ decomposition) occurs at 105 °C and the endothermic peak (Li_3_AlH_6_ decomposition) occurs at 196 °C. Implying that the melt of LiAlH_4_ is inhibited after the addition of the nanosized CoTiO_3_, the LiAlH_4_ + 10 wt.% CoTiO_3_ sample begins to decompose prior to melting. These observations are consistent with the previous reports [35,36].

**Figure 4:**
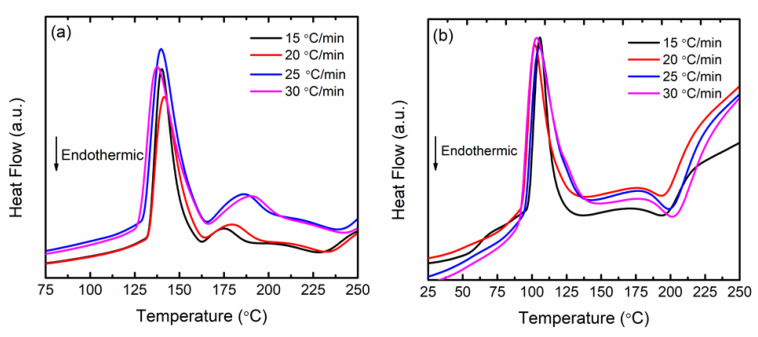
DSC profile of (**a**) as-milled LiAlH_4_ and (**b**) LiAlH_4_ + 10 wt.% CoTiO_3_ at different heating rates.

To scrutinise the impact of nanosized CoTiO_3_ addition on the desorption activation energy of LiAlH_4_, the DSC measurement was performed at several heating rates. The DSC profiles of as-milled LiAlH_4_ and LiAlH_4_ + 10 wt.% CoTiO_3_ at several heating rates are shown in Figure 4. As depicted in the figure, the decomposition peak of LiAlH_4_ + 10 wt.% CoTiO_3_ occurs at a lower temperature than that of undoped LiAlH_4_. The apparent activation energy (*E_A_*) could be fitted from the DSC curve and calculated by Kissinger analysis as follows:

ln[*β*/*T_p_*^2^] = −*E_A_*/*RT_p_* + *A*, (4)

where *β* is the heating rate, *T_p_* is the endothermic peak related to the decomposition temperature, *E_A_* is the activation energy, *R* is the gas constant and *A* is a linear constant.

**Figure 5:**
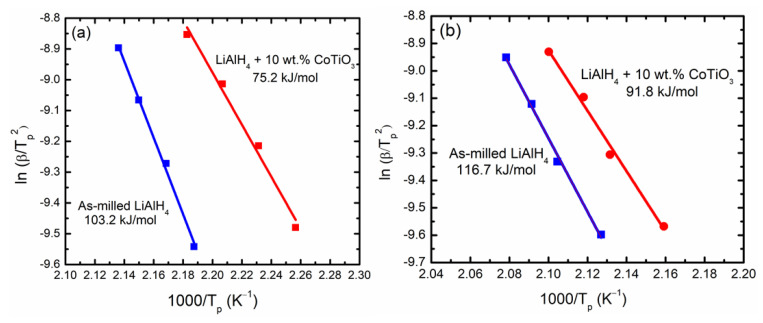
Kissinger plot of (**a**) first and (**b**) second desorption stage of as-milled LiAlH_4_ and LiAlH_4_ + 10 wt.% CoTiO_3_.

Then, the activation energy is determined based on the Kissinger plot of ln [*β*/*T_p_*^2^] versus 1000/*T_p_*, as in Figure 5. The activation energy of the undoped LiAlH_4_ for the first and second desorption stage are 103.2 and 116.7 kJ/mol, respectively, based on the Kissinger plot. By adding nanosized CoTiO_3_, the activation energy of LiAlH_4_ was notably decreased to 75.2 and 91.8 kJ/mol for the first and second desorption stage, respectively, being lowered by 27% for the first stage and 21% for the second stage compared to undoped LiAlH_4_. The reduced activation energy was in good agreement with the reduced decomposition temperature. From these results, it can be clearly seen that the introduction of CoTiO_3_ as an additive remarkably reduced the activation energy of LiAlH_4_, demonstrating that CoTiO_3_ has a favourable impact on the desorption behaviours of LiAlH_4_.

**Figure 6:**
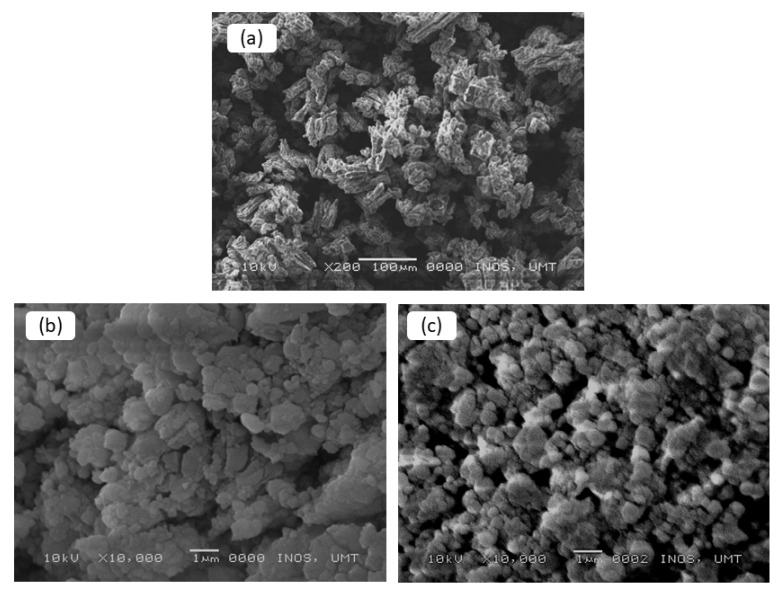
SEM images of (**a**) commercial LiAlH_4_, (**b**) as-milled LiAlH_4_ and (**c**) LiAlH_4_ + 10 wt.% CoTiO_3_ sample.

Figure 6 displays the SEM micrograph of commercial LiAlH_4_, as-milled LiAlH_4_ and LiAlH_4_ + 10 wt.% CoTiO_3_ sample. By referring to Figure 6a, commercial LiAlH_4_ consists of large uniform and blocky-shaped particles. After undergoing the milling process of one hour (Figure 6b), the blocky shape was broken into non-uniform smaller particles, but with some agglomerations. Meanwhile, after the addition of CoTiO_3_, as shown in Figure 6c, the particle was transformed to a finer shape and size. The morphology of the composite sample (CoTiO_3_-doped LiAlH_4_) presents more uniform distributions with fewer agglomerations. The difference in morphology between the milled LiAlH_4_ and LiAlH_4_ + 10 wt.% CoTiO_3_ sample may be due to the presence of an additive that acts as a lubricant and prevents the agglomerations of the sample. These superfine particles could facilitate the rapid hydrogen desorption of LiAlH_4_ [32]. As reported previously, metal oxide-based additives have shown good lubricating performance [37,38]. This indicates that the addition of CoTiO_3_ is favourable in constraining the agglomeration of the LiAlH_4_ and results in a smaller and finer shape of the particle.

**Figure 7:**
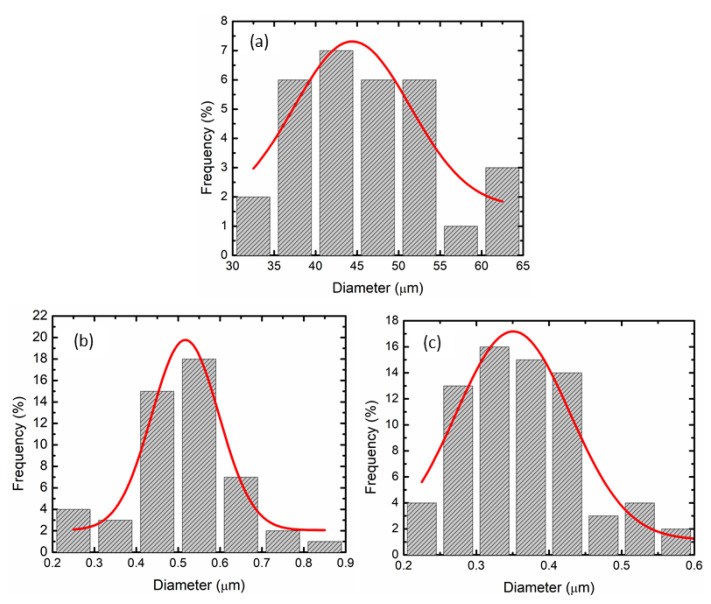
The particle size distribution of (**a**) commercial LiAlH_4_, (**b**) as-milled LiAlH_4_ and (**c**) LiAlH_4_ + 10 wt.% CoTiO_3_.

Figure 7 presents the particle distribution size of commercial LiAlH_4_, as-milled LiAlH_4_ and LiAlH_4_ + 10 wt.% CoTiO_3_ sample evaluated by the Image J software. The average particle size of the commercial LiAlH_4_, as-milled LiAlH_4_ and LiAlH_4_ + 10 wt.% CoTiO_3_ sample was calculated to be ~44, 0.5 and 0.3 µm, respectively. The morphological alteration and significant size reduction are responsible for the large surface defects and the expansion of grain boundaries across the composite’s surface [39]. Resulting in more reactions for the nucleation sites and better diffusion channels of hydrogen being achieved with amplified grain boundaries that enhance the desorption behaviour of the LiAlH_4_-CoTiO_3_ sample with rapid desorption kinetics and lower activation energy, accelerating the rate of hydrogen diffusion [40], the reduction in particle size also results in a shorter hydrogen diffusion path. Similarly, other findings also found that smaller and finer particle distributions demonstrated superior dehydriding performance of LiAlH_4_ [36,41].

**Figure 8:**
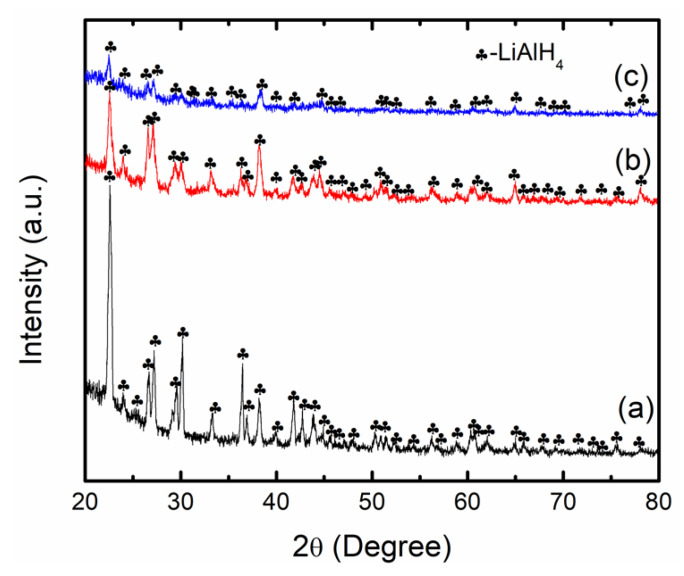
The XRD profile of (a) commercial LiAlH_4_, (b) as-milled LiAlH_4_ and (c) LiAlH_4_ + 10 wt.% CoTiO_3_.

To discover the reaction mechanism during the milling process, XRD analysis was carried out as presented in Figure 8. Figure 8a shows the XRD peaks of commercial LiAlH_4_, and it appears that only peaks of LiAlH_4_ were discovered, indicating the high purity of LiAlH_4_. For the milled sample (Figure 8b), similar to commercial LiAlH_4_, only peaks of LiAlH_4_ were detected, suggesting that, similar to a previous report [42], LiAlH_4_ maintains high stability throughout the milling process. Meanwhile, for the LiAlH_4_ + 10 wt.% CoTiO_3_, as shown in Figure 8c, only LiAlH_4_ peaks were visible, while the peaks of CoTiO_3_ were not identified by the XRD, indicating that the amount of CoTiO_3_ was too small to be detected by the XRD. This phenomenon was comparable to a prior work where several additives such as Ti_3_C_2_ and FeCl_2_ were not detected by XRD [2,43].

**Figure 9:**
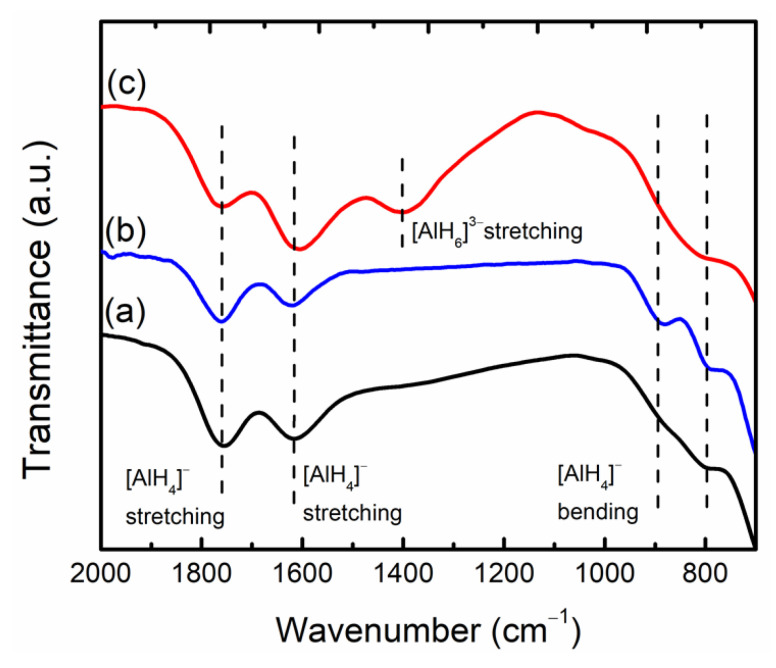
The FTIR profile of (a) commercial LiAlH_4_, (b) as-milled LiAlH_4_ and (c) LiAlH_4_ + 10 wt.% CoTiO_3_.

Figure 9 depicts the FTIR spectra of commercial LiAlH_4_, as-milled LiAlH_4_ and LiAlH_4_ + 10 wt.% CoTiO_3_ sample. FTIR analysis was performed to determine the existence of Li_3_AlH_6_ after the milling process. All three samples exhibit two distinct region modes around 800–900 cm^−1^, corresponding to the Li-Al-H bending mode, and 1600–1800 cm^−1^, attributed to the Al-H stretching mode. However, for the LiAlH_4_ + 10 wt.% CoTiO_3_ sample (Figure 9c), an extra absorbance peak around 1400 cm^−1^ was identified, indicating the presence of the Al-H stretching mode corresponding to the Li_3_AlH_6_. This result proves that LiAlH_4_ was slightly decomposed to Li_3_AlH_6_ during the milling process of LiAlH_4_ and CoTiO_3_.

**Figure 10:**
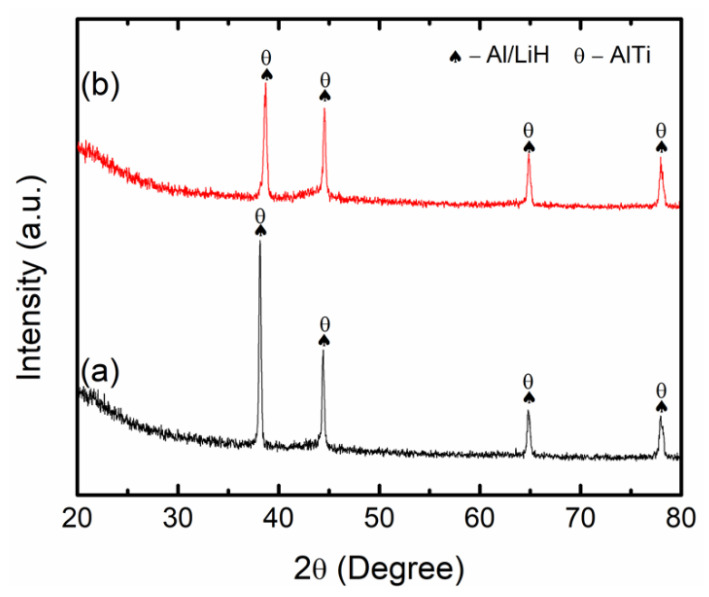
The XRD profile of (a) LiAlH_4_ + 10 wt.% CoTiO_3_ and (b) LiAlH_4_ + 20 wt.% CoTiO_3_ after desorption at 250 °C.

To explore the specific mechanism and catalytic activity of CoTiO_3_ that contributed to the superior desorption behaviours of LiAlH_4_, the 10 wt.% and 20 wt.% CoTiO_3_-doped LiAlH_4_ samples after desorption at 250 °C were analysed using XRD, as depicted in Figure 10. After the desorption process of the 10 wt.% CoTiO_3_-doped LiAlH_4_ sample (Figure 10a), the main peaks detected are the dehydrogenated products of LiAlH_4_, which are LiH and Al, denoting that the complete desorption process of LiAlH_4_ occurs. Moreover, the peaks of AlTi were also detected, indicating that there is a reaction between LiAlH_4_ and CoTiO_3_. However, the peaks of Co or Co-containing species were not identified. The additional characterisation was carried out with 20 wt.% of CoTiO_3_ (Figure 10b). Similarly, the main peaks of LiH and Al were observed with the additional peak of AlTi, and the fact that no peaks of Co or Co-containing species were identified after the desorption process may be due to the amorphous form of Co or Co-containing species.

Referring to the results, it is noteworthy to state that the desorption behaviours of LiAlH_4_ were improved due to the in situ formation of active species during the heating process. In this study, the formation of AlTi and Co or Co-containing species was believed to contribute to the enhanced desorption behaviours of LiAlH_4_. This phenomenon agrees with a previous study that also reported the formation of AlTi after the dehydrogenation process of LiAlH_4_ [3,44]. The in situ formation of AlTi is beneficial in ameliorating the desorption behaviour of LiAlH_4_ by catalysing the dehydrogenation through reaction in Equations (1) and (2). In addition, the study performed by Wohlwend et al. [45] revealed the superior performance of LiAlH_4_ with the addition of Ti-based additives. The Ti atom reacts strongly with the LiAlH_4_ surface and reduces the H binding energy. The reduction in binding energy may be a result of the correlation of the charge transfer change between Al and H that results in the accelerated kinetic performance of LiAlH_4_. It denotes that Co or Co-containing species also occupy a dominant role in improving the desorption behaviour of LiAlH_4_, even though the Co or Co-containing phase was not detected. For instance, a previous study discovered that the presence of Co_2_O_3_ exhibited a significant enhancement in the desorption of LiAlH_4_ compared to undoped LiAlH_4_ [46]. Li et al. [29] also indicated that the formation of Co-containing species after the desorption process of LiAlH_4_ provides a positive impact on the desorption behaviour of LiAlH_4_. Therefore, it is reasonable to infer that the formation of these active species contributes to the notable advancement of the desorption behaviour of LiAlH_4_. By shortening the length of diffusion of the reaction ions [29,41], these in situ-formed AlTi and Co or Co-containing species are responsible for boosting the desorption behaviour of LiAlH_4_ by taking part as the active sites for the nucleation and creation of the dehydrogenation yield. However, further characterisation using X-ray photoelectron spectroscopy and transmission electron microscopy is needed to evaluate the actual impact and mechanism of the LiAlH_4_-CoTiO_3_ system.

## 4. Conclusions

The introduction of nanosized CoTiO_3_ that was synthesised via a solid-state method significantly ameliorates the desorption behaviour of LiAlH_4_. The LiAlH_4_-CoTiO_3_ sample began to release hydrogen at around 115–120 °C and 144–150 °C for the first and second desorption stages, respectively, lower than for undoped LiAlH_4_. The addition of nanosized CoTiO_3_ also results in accelerated desorption kinetics with the ability to desorb hydrogen 30–35 times faster than undoped LiAlH_4_. The rapid hydrogen desorption performance of LiAlH_4_ when added with CoTiO_3_ may be due to the lower activation energy of the LiAlH_4_-CoTiO_3_ sample, which was calculated to be 75.2 kJ/mol (first stage) and 91.8 kJ/mol (second stage), reduced by 28.0 and 24.9 kJ/mol compared to undoped LiAlH_4_. The nanosized CoTiO_3_ was also beneficial in the reduction of LiAlH_4_ particle size during the milling process. Smaller particle size is favourable in reducing the hydrogen diffusion length, which then results in superior desorption performance. The enhanced desorption behaviour of LiAlH_4_ was also due to the synergetic effect of the in situ formation of AlTi and Co or Co-containing species during the heating process of LiAlH_4_ and CoTiO_3_. By reducing the initial desorption temperature and activation energy, the addition of nanosized CoTiO_3_ remarkably ameliorates the desorption behaviour of LiAlH_4_, resulting in finer and smaller particles and accelerating the desorption kinetic behaviour. These findings shed light on the preparation of LiAlH_4_ hydrogen storage systems for mobile applications.

## Data Availability

The data presented in this study are available on request from the corresponding author.
